# p53 mitotic centrosome localization preserves centrosome integrity and works as sensor for the mitotic surveillance pathway

**DOI:** 10.1038/s41419-019-2076-1

**Published:** 2019-11-07

**Authors:** Claudia Contadini, Laura Monteonofrio, Ilaria Virdia, Andrea Prodosmo, Davide Valente, Luciana Chessa, Antonio Musio, Luca L. Fava, Cinzia Rinaldo, Giuliana Di Rocco, Silvia Soddu

**Affiliations:** 1 Unit of Cellular Networks and Molecular Therapeutic Targets, IRCCS-Regina Elena National Cancer Institute, Rome, Italy; 2 Department of Clinical and Molecular Medicine, Sapienza University, Rome, Italy; 3 Institute of Genetics and Biomedical Research, National Research Council (CNR), Pisa, Italy; 4 Armenise-Harvard Laboratory of Cell Division, Department of Cellular, Computational and Integrative Biology - CIBIO, University of Trento, Povo, Italy; 5 Institute of Molecular Biology and Pathology, National Research Council (CNR), c/o Sapienza University, Rome, Italy; 6 Present Address: Department of Biology, University of Rome “Tor Vergata”, 00133 Rome, Italy; 7 Present Address: Laboratory of Cardiovascular Science, NIA/NIH Baltimore, Baltimore, MD 21224 USA; 8 Present Address: GMP Biopharmaceutical Facility, Ospedale Pediatrico Bambino Gesù, Rome, Italy

**Keywords:** Tumour-suppressor proteins, Checkpoints

## Abstract

Centrosomal p53 has been described for three decades but its role is still unclear. We previously reported that, in proliferating human cells, p53 transiently moves to centrosomes at each mitosis. Such p53 mitotic centrosome localization (p53-MCL) occurs independently from DNA damage but requires ATM-mediated p53Ser15 phosphorylation (p53Ser15^P^) on discrete cytoplasmic p53 foci that, through MT dynamics, move to centrosomes during the mitotic spindle formation. Here, we show that inhibition of p53-MCL, obtained by p53 depletion or selective impairment of p53 centrosomal localization, induces centrosome fragmentation in human nontransformed cells. In contrast, tumor cells or mouse cells tolerate p53 depletion, as expected, and p53-MCL inhibition. Such tumor- and species-specific behavior of centrosomal p53 resembles that of the recently identified sensor of centrosome-loss, whose activation triggers the mitotic surveillance pathway in human nontransformed cells but not in tumor cells or mouse cells. The mitotic surveillance pathway prevents the growth of human cells with increased chance of making mitotic errors and accumulating numeral chromosome defects. Thus, we evaluated whether p53-MCL could work as a centrosome-loss sensor and contribute to the activation of the mitotic surveillance pathway. We provide evidence that centrosome-loss triggered by PLK4 inhibition makes p53 orphan of its mitotic dock and promotes accumulation of discrete p53Ser15^P^ foci. These p53 foci are required for the recruitment of 53BP1, a key effector of the mitotic surveillance pathway. Consistently, cells from patients with constitutive impairment of p53-MCL, such as ATM- and PCNT-mutant carriers, accumulate numeral chromosome defects. These findings indicate that, in nontransformed human cells, centrosomal p53 contributes to safeguard genome integrity by working as sensor for the mitotic surveillance pathway.

## Introduction

Numeral and structural defects of the centrosomes are prevalent in cancer and are thought to contribute to tumorigenesis by promoting abnormal mitotic spindles and chromosomal instability^1^ or increasing tumor cells invasiveness^2^. Besides the well-established role as “guardian of the genome”^3,4^, the p53 tumor suppressor has been proposed as “guardian of ploidy” acting in the prevention of structural and numeral centrosome alterations through its transcription function^5–13^. Recently, a mitotic surveillance pathway has been identified upon genetic or chemical inhibition of the centrosome assembly initiator, PLK-4^14–16^. It has been shown that in nontransformed human cells, but not mouse cells or tumor cells, centrosome-loss or prolonged mitosis trigger cell-cycle arrest through a non-canonical 53BP1/USP28-p53-p21^WAF1^ axis^17–19^. This pathway is thought to prevent the growth of cells that have an increased chance of making mitotic errors. However, the molecular sensor linking centrosome-loss or prolonged mitosis and the 53BP1-mediated p53 activation is still undefined.

**Fig. 1:**
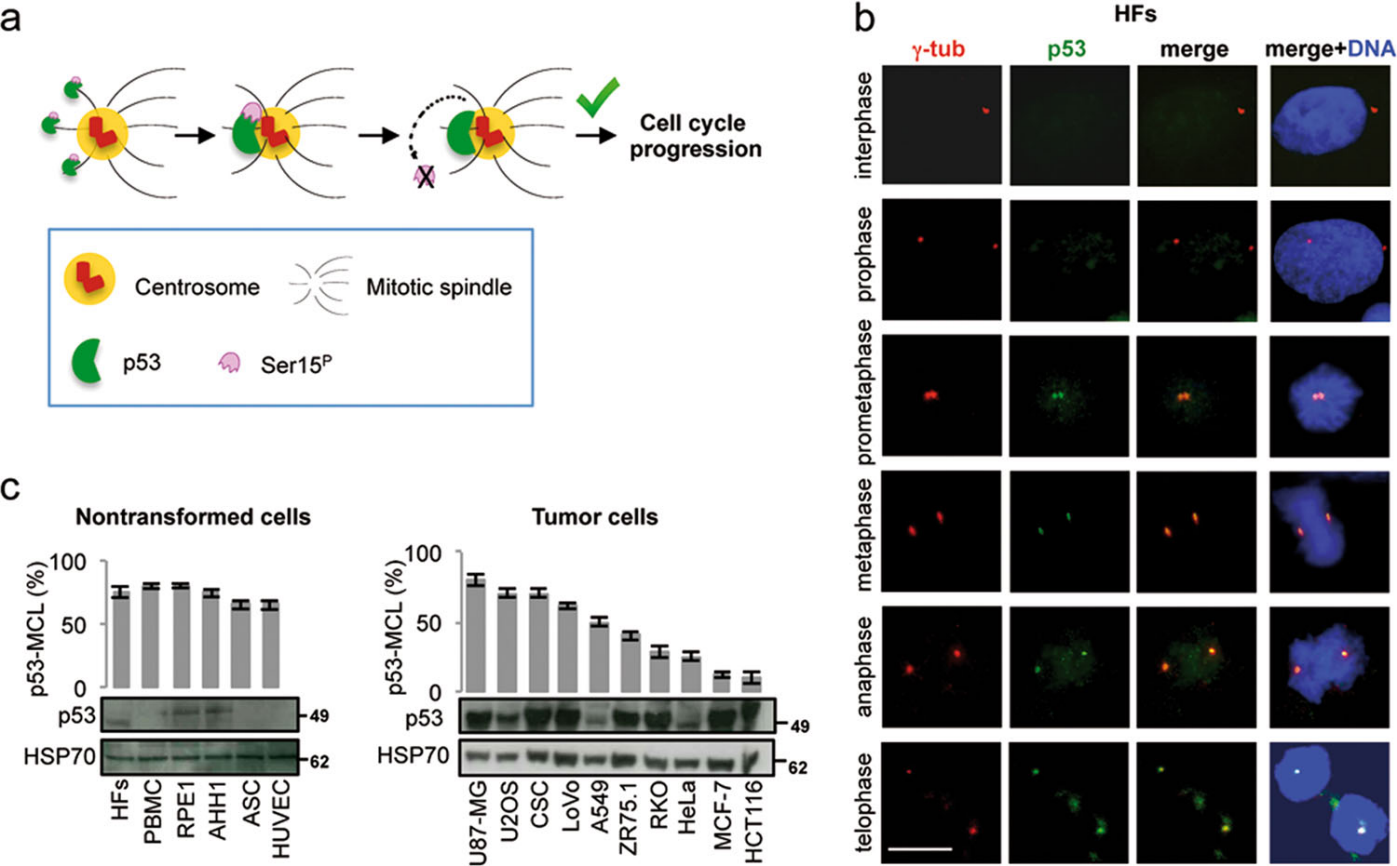
p53-MCL in human cells. **a** Schematic representation of p53-MCL as previously described^23^. At each mitosis during spindle formation, p53 is phosphorylated at Ser15 by ATM and, through MTs moves to centrosomes where it is suddenly dephosphorylated to allow cell-cycle progression. Only one of the two spindle pole is represented. **b** Proliferating, unsynchronized human immortalized fibroblasts (HFs) were fixed and immunostained for the indicated proteins. DNA was stained with HOECHST-33342 to identify mitoses. Representative images of the indicated phases of the cell cycle show that endogenous p53 colocalizes with γ-tubulin from prometaphase to telophase, but not in interphase (none out of > 500 interphases analyzed). **c** Proliferating, unsynchronized cells of the indicated lines were grown on coverslips, fixed, and stained as in (**b**). For each coverslip, > 200 mitotic cells (*n* > 200 mitoses) were analyzed to measure p53-MCL (i.e., the percentage of mitotic cells in which endogenous p53 colocalizes with γ-tubulin at both centrosomes). Histograms show the percentage of p53-MCL in a series of human nontransformed and tumor cells. Below histograms are the relative immunoblots performed on total cell extracts (TCEs) from the indicated cells to evaluate their p53 amount. Scale bars, 10 µm

In addition to the well-characterized transcriptional and mitochondrial activities, centrosome localization of p53 has been described for three decades in chick, rodent, and human cells^20–24^. However, the functional role of p53 in this specific subcellular localization is still unknown. A contribution of centrosomal p53 in the control of centrosome duplication in mouse cells has been proposed but it has never been confirmed in human cells^25–28^. We have previously shown that, in human hemopoietic cells, at each mitosis p53 transiently moves to centrosomes in ATM- and microtubule (MT)-dependent manner^23,29^. ATM phosphorylates p53Ser15 on discrete cytoplasmic p53 foci that move to centrosomes by MT dynamics. In unperturbed mitotic cells, once reached the centrosomes, p53Ser15^P^ is suddenly dephosphorylated and the cell-cycle progress (Fig. 1a)^23,30,31^. This ATM-dependent centrosomal localization of p53 is so consistent during the cell cycle as to allow us to develop a functional test to identify individuals carrying mutations in the *ATM* gene^29^. In particular, by measuring the percentage of mitotic cells in which p53 colocalizes with the centrosomes in lymphoblastoid cell lines (LCLs) and in cell cycle-reactivated peripheral blood mononuclear cells (PBMCs), we have been able to discriminate healthy individuals (i.e., wild-type ATM alleles; p53-MCL > 75%) from Ataxia-Telangiectasia (A-T) patients (i.e., biallelic ATM mutations; p53-MCL < 30%) and from A-T healthy carriers (i.e., monoallelic ATM mutations; p53-MCL > 40% < 60%)^29,32^. However, which is the function of the ATM-p53 axis at the centrosome is still unclear. Here we show that inhibition of p53-MCL results in centrosome fragmentation and cell death in nontransformed human cells, but not in mouse cells and tumor cells, and that centrosomal p53 works as sensor for the mitotic surveillance pathway.

## Results

### p53-MCL is present in nontransformed human cells of different histotype

We previously described p53-MCL in hemopoietic LCLs and PBMCs by double immunofluorescence (IF) with anti-p53 and anti-γ-tubulin antibodies (Abs)^23,29,30^. To investigate the functional role of centrosomal p53, we first verified whether p53-MCL can be also detected in cells from different tissues. IF analyses of hTERT-immortalized human fibroblasts (HFs) (Fig. 1b) confirmed the presence of p53 at the centrosomes in different mitotic phases (i.e., from prometaphase to telophase, excluding prophase), while no centrosomal p53 staining was observed in interphase. Thus, p53-MCL was measured by calculating the percentage of mitotic cells—from prometaphase to telophase—with p53 localized at both centrosomes^29^. This p53-MCL estimation was made in a series of primary, immortalized, and tumor cells carrying endogenous wild-type p53 (wt-p53) (Fig. 1c). We found that all the analyzed human primary (i.e., PBMCs, adipose derived stromal cells-ASC, human umbilical vein endothelial cells-HUVEC) and immortalized cells (i.e., HFs, LCLs, retinal pigmented epithelial cells-RPE1) have percentages of p53-MCL > 75%, which are in the range we previously reported for LCLs and PBMCs of healthy donors^29^ (Fig. 1c, left panel). In contrast, human tumor cells showed that, independently of their levels of p53 expression and the *ATM* gene status—that is mutated only in the RKO cells—the percentages of p53-MCL ranged from >75% to <10% (Fig. 1c, right panel). These results indicate that p53 localizes at the centrosomes in mitosis in nontransformed human cells of different histotype while tumor cells can lose this subcellular localization.

### Acute depletion of p53 induces centrosome fragmentation in nontransformed human cells

**Fig. 2:**
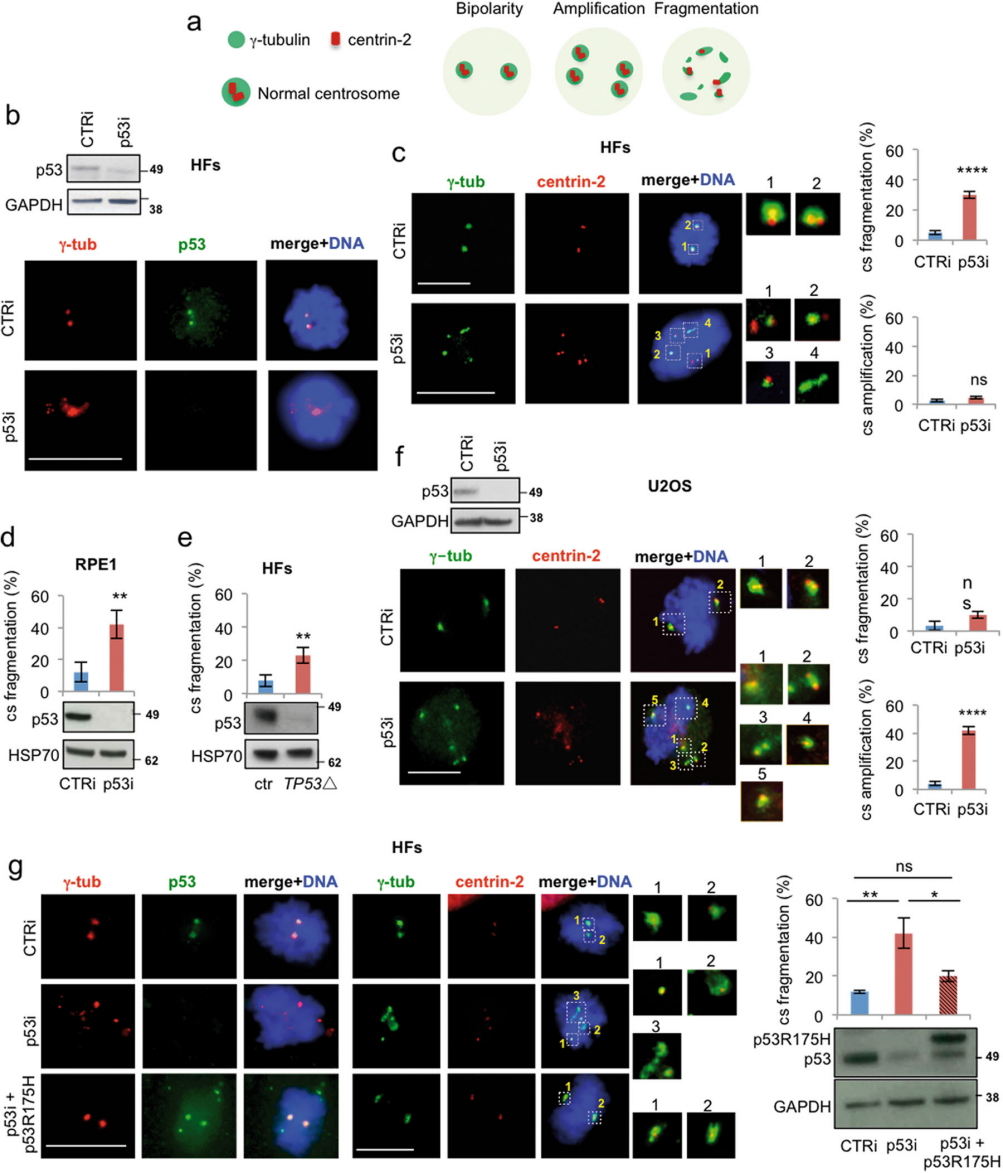
Acute deletion of p53 induces centrosome fragmentation in human nontransformed cells. **a** Schematic representation of centrosome conditions (i.e., physiological bipolarity and pathological amplification and fragmentation) as they can be detected by double IF with anti-γ-tubulin and anti-centrin-2 Abs. **b** Endogenous p53 expression was depleted by p53 siRNA (p53i) in nontransformed HFs. Control (CTRi) and p53i cells were analyzed 48 h post-transfection by WB and double IF for γ-tubulin/p53. **c** The same cells reported in (**b**) were analyzed for centrosome number and structure by IF for γ-tubulin and centrin-2. Representative images with enlarged centrosomes or centrosomal material are reported. Quantifications of the IF images (*n* = 600 mitoses) are reported in the histograms. No significant accumulation of cells with numeral centrosomal defects (cs amplification) is observed. In contrast, strongly significant accumulation of mitoses with centrosome fragmentation is observed in p53i HFs. **d** RPE1 cells were transfected with CTRi and p53i and analyzed as the HFs described in (b and c). WB analysis and quantification of centrosome fragmentation are reported. **e** Endogenous p53 expression was depleted in HFs by transient CRISPR/Cas9 transfection (*TP53*Δ) and analyzed as in (**b**) and (**c**). WB and quantification of centrosome fragmentation are reported. **f** U2OS cells were transfected and analyzed as in (**b**) and (**c**) (*n* = 400 mitoses). In contrast to nontransformed HFs, U2OS tumor cells show centrosome amplification with no centrosome fragmentation. **g** CTRi and p53i HFs were transfected with the transcription-defective p53R175H mutant and p53 protein expression analyzed by WB on TCEs. Cells from the same experiments were fixed and analyzed by double IF for γ-tubulin/p53 and γ-tubulin/centrin-2 (*n* = 300 mitoses). Representative IF images and quantification of centrosome fragmentation are reported. Data show that p53R175H localizes at the centrosomes and significantly reduces their fragmentation in p53i HFs. No effects, at the centrosome level, were induced by p53R175H expression in CTRi HFs. Each experiment was repeated at least three times. Scale bars, 10 µm. ns = not significant; *P < 0.05; **P < 0.01; ***P < 0.001; ****P < 0.0001

Next, we attempted to inhibit p53-MCL through different independent strategies and analyzed the effects on centrosome number and structure by double IF for γ-tubulin and centrin-2 (Fig. 2a). As a first strategy, we induced depletion of p53 by RNA interference with p53-specific siRNAs in HFs cells. p53 depletion was assessed by western blotting (WB) and IF (Fig. 2b) and confirmed by the functional impairment of p53 activation in DNA-damage response (DDR) (Supplementary Fig. 1a). Compared with controls (CTRi), p53-interfered (p53i) HFs showed a significant induction of centrosome fragmentation, as indicated by the accumulation of cells with > 2 γ-tubulin spots, each with one, two, or without centrin-2 spots (Fig. 2c), while no sign of centrosome amplification was observed. Similar results were obtained by a different human nontransformed cell line, the RPE1 (Fig. 2d and Supplementary Fig. 1b). Moreover, acute p53 depletion by transient CRISPR/Cas9 transfection (*TP53*Δ) in HFs produced results comparable to those obtained with siRNA (Fig. 2e and Supplementary Fig. 1c), thus ruling out cell-type specific outcomes and off-target effects. In contrast, when p53 was depleted in human tumor cells such as U2OS cells, no sign of centrosome fragmentation was detected (Fig. 2f). Instead, consistent with previous data^6^, p53i U2OS cells showed centrosome amplification as indicated by the presence of cells with >2 γ-tubulin spots with normal centrin-2 (Fig. 2f). These results were independent from the original levels of p53-MCL (Fig. 1b), since p53i failed to induce centrosome fragmentations (none out of 100 mitoses and Supplementary Fig. 1d) in both HeLa and U87MG tumor cells, which display 20% and 80% of p53-MCL, respectively.

Next, we evaluated whether re-expression of p53 in p53i HFs is able to counteract the centrosome fragmentation induced by p53 depletion. We previously observed that LCLs from Li-Fraumeni patients, which are heterozygous *TP53* mutants, have normal p53-MCL^29^. Thus, to avoid cell-cycle arrest induced by exogenous wt-p53 expression and consequent disappearance of mitotic cells, we used the transcription-defective p53R175H mutant which maintains the ability to localize at the centrosome in mitosis (Fig. 2g and Supplementary Fig. 1e). In p53i HFs, p53R175H expression reduced centrosome fragmentation (Fig. 2g), further excluding off-target effects and indicating that the transcriptional activity of p53 is not required for preserving centrosome integrity.

### Centrosome fragmentation is linked to impaired p53-MCL

**Fig. 3:**
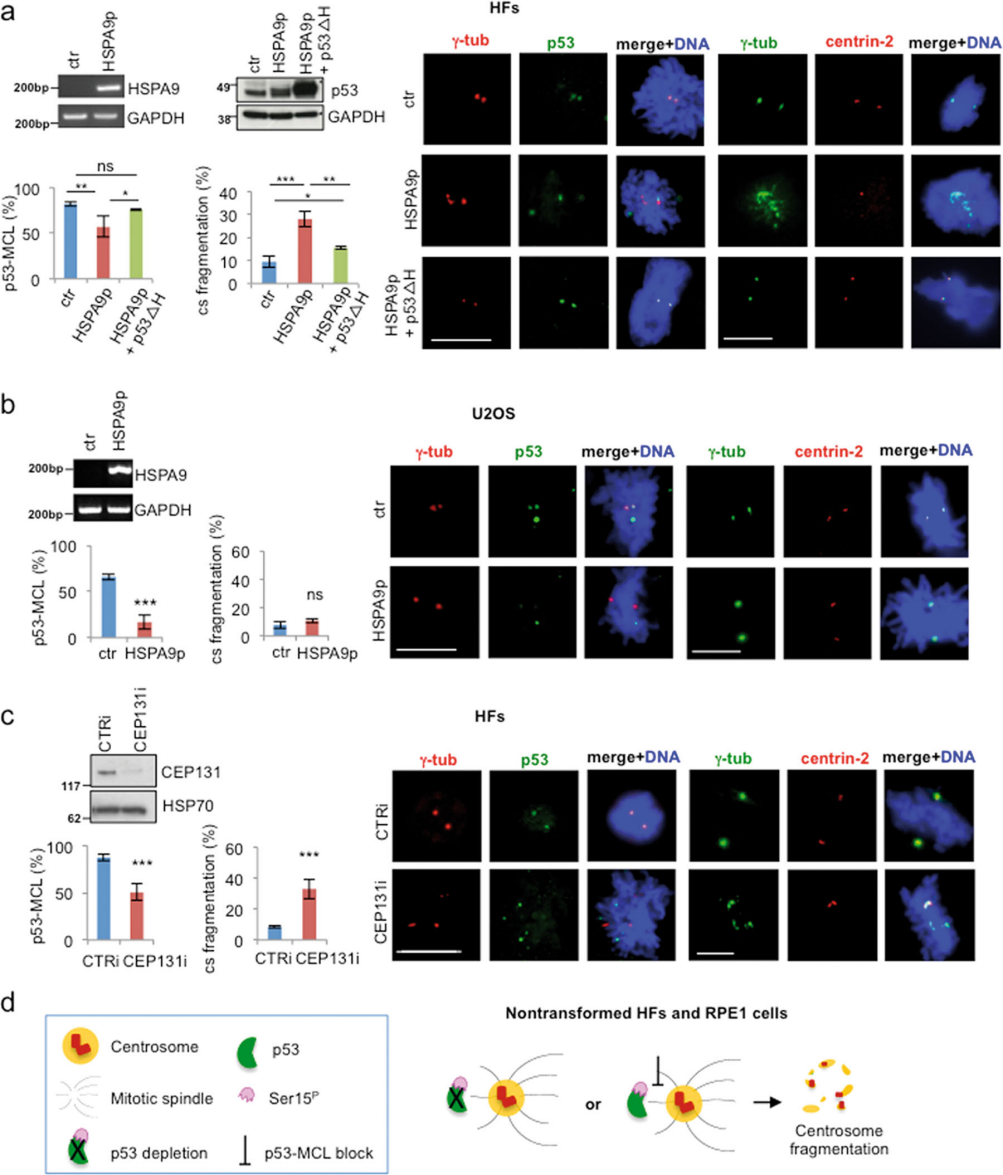
Centrosome fragmentation is linked to impaired p53-MCL. **a** HFs were transfected with the empty vector (ctr), the vector expressing the HSPA9^253–282^ peptide (HSPA9p) and analyzed by semiquantitative RT-PCR since no Abs are available for the detection of the small peptide. In addition, HFs were co-transfected with the HSPA9p-expressing vector and a second vector encoding for human p53-Δ(325–355), missing the region required to bind HSPA9 protein (p53ΔH) and analyzed by WB. p53-MCL was analyzed by double IF for γ-tubulin/p53 and centrosome structure by double IF for γ-tubulin/centrin-2, as described in Fig. 2. Representative IF images and histograms show that HSPA9p expression impairs p53-MCL and induces centrosome fragmentation. At variance from p53i HFs, in these conditions, p53 is still present but does not colocalize with γ-tubulin. When p53-MCL was restored by p53ΔH expression, centrosome fragmentation was inhibited, as shown by representative IF images and relative histograms. These experiments were repeated three times and at least 200 mitoses per sample were analyzed. **b** U2OS cells were transfected with the empty vector (ctr) and the vector expressing the HSPA9p and analyzed as described in (**a**). In these cells, p53-MCL was even more significantly reduced than in HFs, but no sign of centrosome fragmentation was detected. These experiments were repeated three times and 300 mitoses per sample were analyzed. **c** HFs were transfected with CTRi and CEP131i siRNAs, analyzed for CEP131 expression by WB and for p53-MCL and centrosome structure and number as above. Representative IF images and histograms show that CEP131-depletion inhibits p53-MCL and induces centrosome fragmentation. These experiments were repeated four times and at least 200 mitoses per sample analyzed. ns = not significant; *P < 0.05; **P < 0.01; ***P < 0.001. Scale bars, 10 µm. **d** Schematic representation of the results obtained in nontransformed human cells by blocking p53-MCL (i.e., p53 depletion or impairment of p53 centrosomal localization)

To provide more direct evidence that centrosome fragmentation is related to the impairment of p53-MCL rather than loss of p53 expression, we inhibited p53-MCL without altering the total amount of p53, through two additional independent strategies. First, we used a small peptide corresponding to amino acids 253–282 of HSPA9, the human heat shock protein family A (Hsp70) member 9, that binds p53 and prevents its centrosome localization without affecting p53 transactivation function and protein stability^33^. Similarly to p53i, transient expression of the HSPA9^253–282^ peptide (HSPA9p) impaired p53-MCL in both HFs and U2OS cells but induced centrosome fragmentation only in the HFs (Fig. 3a, b).

Next, we attempted to rescue centrosome integrity in HSPA9p-transfected cells by expressing a p53 protein lacking the HSPA9-binding region (i.e., amino acids 325–355; p53ΔH), that has been reported to localize at the centrosome also in cells overexpressing HSPA9^33^. Consistent with previous data, we found that p53ΔH localizes at the centrosome in >70% of the cells, independently of the presence of HSPA9p expression (Supplementary Fig. 2a), and showed that it suppresses the centrosome fragmentation induced by HSPA9p (Fig. 3a).

As further strategy of p53-MCL inhibition, we gained clues on factors that might recruit p53 at the centrosomes by restoring p53-MCL in LCLs from A-T heterozygous (A-T htz) carriers. In these cells, we attempted to increase wild-type ATM (ATM-wt) expression and subsequent p53-MCL through 5-azacytidine-induced DNA demethylation^34^. We observed a recovery of p53-MCL comparable to those of LCLs from healthy donors that, however, was not associated with an increase of ATM protein levels and/or activity, but rather with the induction of azacytidine-induced protein 1, also known as CEP131 (Supplementary Fig. 2b). CEP131 is a centriolar satellite protein recruited by pericentrin and involved in cilium formation, centrosomal remodeling, and prevention of chromosomal instability in tumor cells^35–38^. In HFs, CEP131-depletion by RNAi (CEP131i) reduced p53-MCL and induced centrosome fragmentation without affecting the DDR activity of p53 (Fig. 3c and Supplementary Fig. 2c). In contrast, in U2OS tumor cells, CEP131i induced no sign of centrosome fragmentation but only a mild centrosome amplification (7.5% in CTRi vs. 15% in CEP131i) as previously reported^36^. These results show that inhibition of p53-MCL induces centrosome fragmentation in nontransformed human cells but not in tumor cells (Fig. 3d).

### p53 centrosomal localization is different in human vs. mouse cells

**Fig. 4:**
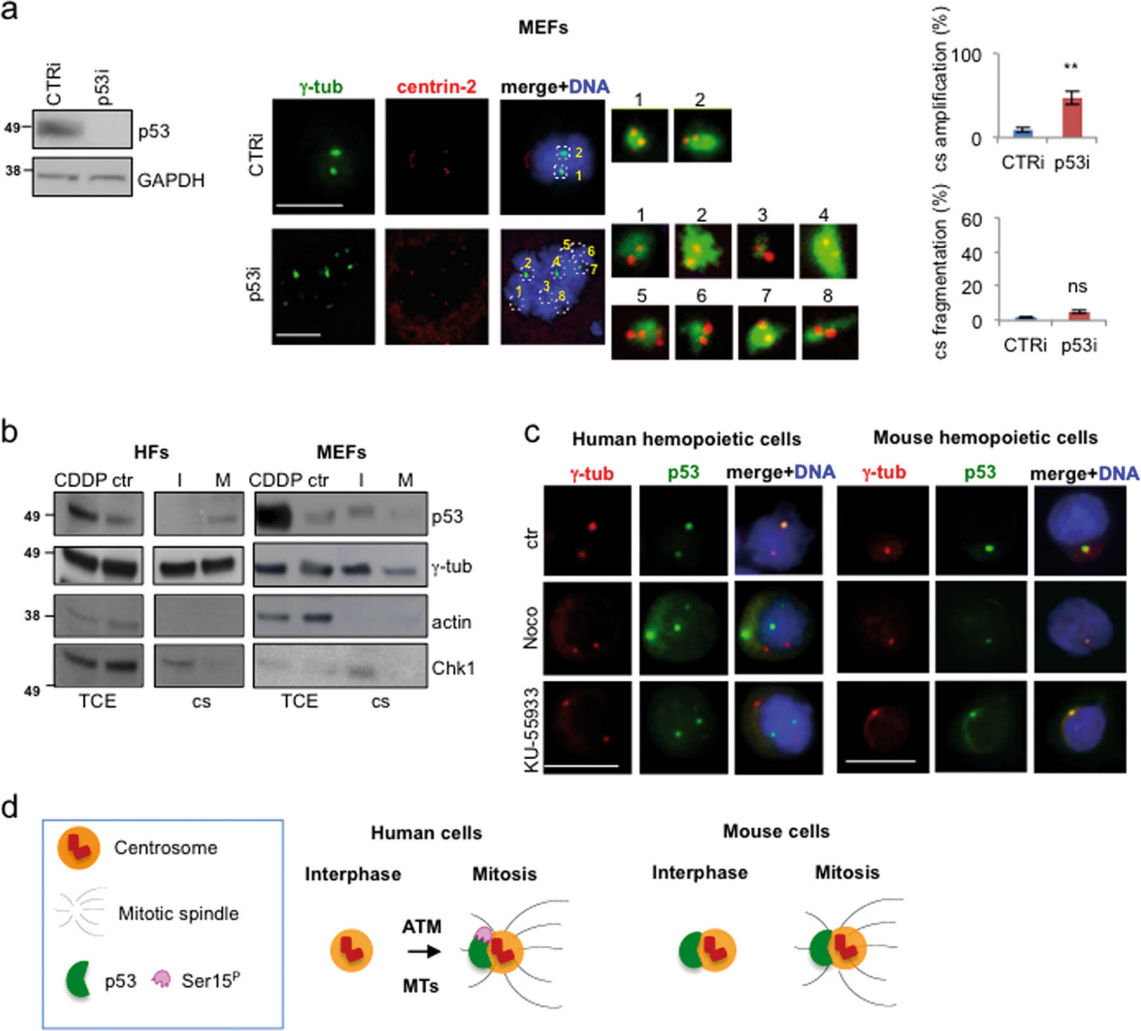
p53 centrosomal localization in mouse cells. **a** MEFs were transfected with CTRi and p53i-specific siRNAs and analyzed by double IF for γ-tubulin/centrin-2 to evaluated centrosome number and structure as described above for human cells. WB analysis of TCEs shows depletion of p53 protein level. Representative IF images with enlarged centrosomes on the right show the normal structure of the centrosomes. Histograms show the percentage of p53i MEFs (*n* = 600) with centrosome amplification, compared to CTRi cells (*n* = 400). In contrast to human nontransformed cells, no centrosome fragmentation is detected. This experiment was repeated twice in duplicate. ns = not significant. *P < 0.05; **P < 0.01. Scale bars, 10 µm. **b** Immunoblots of the indicated proteins were performed on lysates from isolated centrosomes (cs) purified from interphase (I) and mitotic (M) enriched human (HFs) and mouse (MEFs) fibroblasts. The following controls were performed: (i) TCE of untreated or cisplatin (CDDP)-treated cells were used as p53 detection control; (ii) γ-tubulin was used as constitutive centrosomal marker; (iii) actin, that never localizes at the centrosome, as centrosome purification control; (iv) Chk1, that does not localize at the centrosome in mitosis, was used as purification control of mitotic centrosomes. p53-MCL was confirmed in human cells while mouse p53 is present at the centrosomes both in interphase and mitosis. This experiment was repeated four times. Images of a representative experiment are shown. **c** Representative IF images of human (AHH1) and mouse (32D) hemopoietic cells solvent-treated (ctr) or treated with nocodazole, to inhibit MTs, and KU-55933, to inhibit ATM. In contrast to human cells that were used as positive control (e.g., p53-MCL declines from 79 ± 3 to 15 ± 2% with nocodazole and to 10 ± 4% with KU-55933 in the reported experiment), mouse cells showed a p53 centrosomal localization independent of both MT dynamics (p53/γ-tubulin colocalization = 85 ± 3%) and ATM activity (p53/γ-tubulin colocalization = 83 ± 3%). Experiments were reproduced at least three times in duplicate and at least 200 cells per sample analyzed. Scale bars, 10 µm. **d** Schematic representation of the different centrosomal localization of p53 in human and mouse cells

In mouse embryo fibroblasts (MEFs), p53 deficiency has been shown to induce centrosome amplification, at least in part through p53 centrosome localization^6,7,28^, while, to our knowledge, centrosome fragmentation has not been reported. Thus, we analyzed p53i MEFs by double IF for γ-tubulin and centrin-2 as we did for human cells. Similar to the previously reported data on MEFs from p53^−/−^ mice, p53i MEFs showed centrosome amplification while no sign of centrosome fragmentation was detected (Fig. 4a). This result further rules out nonspecific outcomes of our experimental procedures. However, it opens the question why human and mouse cells show this divergent centrosome response. Thus, we compared the centrosomal localization of p53 in HFs and MEFs by WB analyses of purified centrosomes isolated from interphase and mitosis-enriched cells. As shown in Fig. 4b, HF’s p53 was detected only in the mitotic centrosomes while MEF’s p53 was present both in interphase and mitotic centrosomes suggesting different p53 centrosome localization pattern in human vs. mouse cells. These data were further supported by the divergent results we obtained by inhibiting MT polymerization with nocodazole and ATM activity with KU-55933. These two conditions are known to impair p53-MCL in human cells^23,30^ (Fig. 4c, left panels) but did not modify the p53 centrosome localization in mouse cells (Fig. 4c, right panels), indicating that mouse p53 localizes at the centrosome in ATM- and MT-independent manner. These results show that p53 centrosomal localization is different in human vs. mouse cells (Fig. 4d). Of note, a human-specific, centrosome-associated p53 function has been recently described in response to centrosome-loss and the subsequent activation of the mitotic surveillance pathway^15^.

### Centrosome fragmentation by acute impairment of p53-MCL induces cell death

**Fig. 5:**
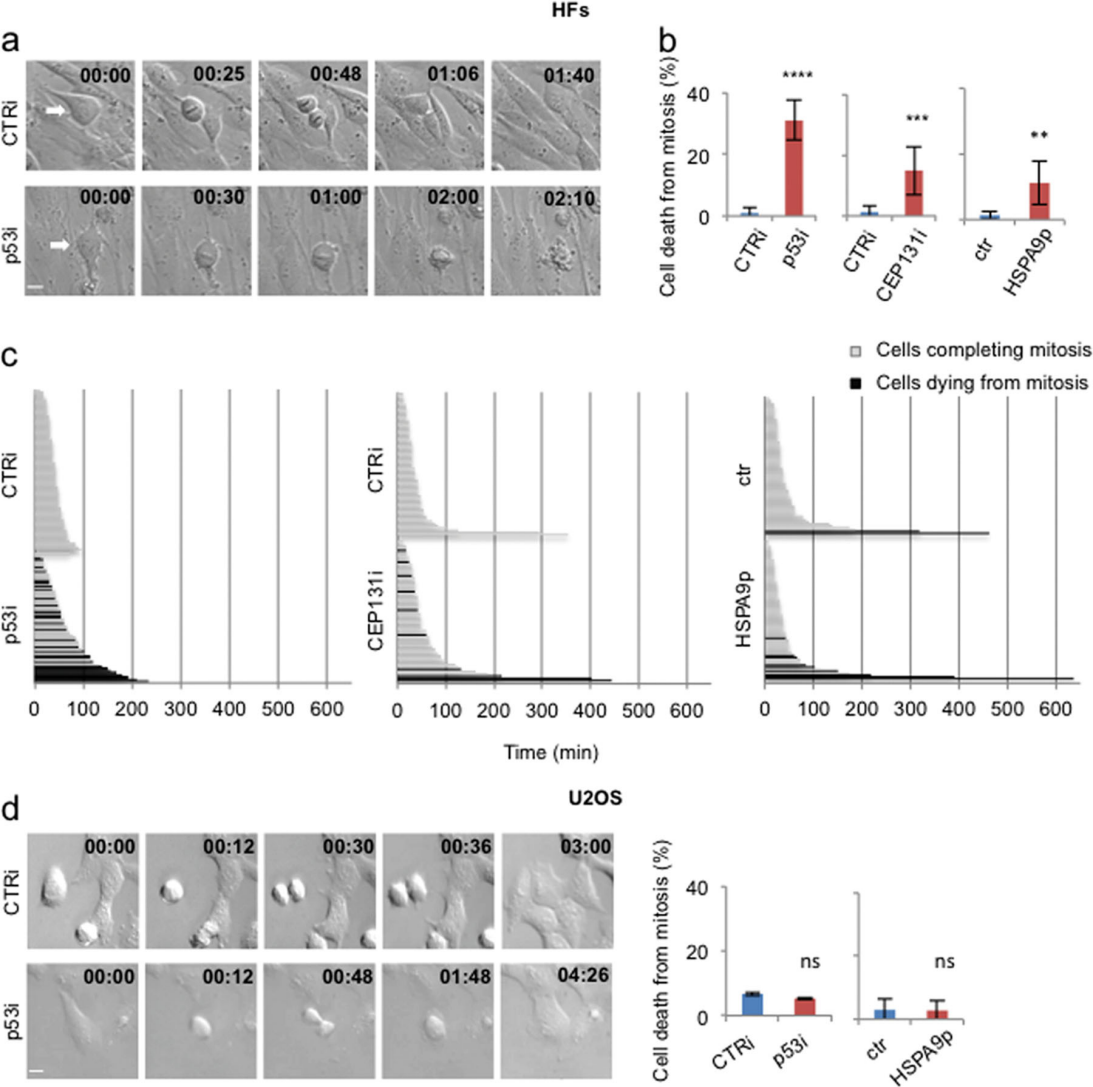
Centrosome fragmentation induced by acute impairment of p53-MCL causes cell death. **a** Representative stills from time-lapse recording of CTRi and p53i HFs. CTRi cells underwent successful mitosis and cytokinesis giving rise to two mononucleated daughter cells, while p53i cells underwent cell death at high frequency. Scale bars, 10 µm. **b** Histograms show the percentages of mitotic cells undergoing cell death during the time-lapse recording, after the different strategies used to reduce p53-MCL, i.e., p53i, CEP131i, and expression of HSPA9p. ***P < 0.001; ****P < 0.0001. **c** Fate profiles show in black the time from mitotic enter to death (i.e., from roundup to death) for the mitotic cells that died during the recording, and in gray the time from mitotic enter to anaphase beginning (i.e., from roundup to cleavage furrow ingression) for cells that complete mitosis. Each line depicts a single cell. Zero time on fate profiles represents when cells entered mitosis. In the left histogram, the median of timing is 42 min in the CTRi cells (*n* = 107) and 57 min in the p53i cells (*n* = 86). In the middle histogram, the median of timing is 30 min in the CTRi cells (*n* = 62) and 42 min in the CEP131i cells (*n* = 62). In the right histogram, the median of timing is 30 min in the ctr cells (empty vector, *n* = 70) and 30 min in the HSPA9p cells (*n* = 63)

To evaluate the consequences of centrosome fragmentation we observed by inhibiting p53-MCL in HFs, we performed time-lapse analyses of mitotic cells. Differently from CTRi HFs, p53i, CEP131i, and HSPA9p cells died in a significant percentage of the analyzed mitosis (Fig. 5a, b and Videos 1–4). In addition, evaluation of the timing from roundup to anaphase or the beginning of cell death showed that a significant proportion of p53i, CEP131i, and HSPA9p HFs died with a delay in the prophase to anaphase transition (Fig. 5c), suggesting that cell death might be, at least in part, related to mitotic delay. Finally, in agreement with the absence of centrosome fragmentation, when time-lapses were performed in U2OS cells, no significant sign of cell death was detected (Videos 5–7 and Fig. 5d).

### p53-MCL works as a centrosome-associated sensor

**Fig. 6:**
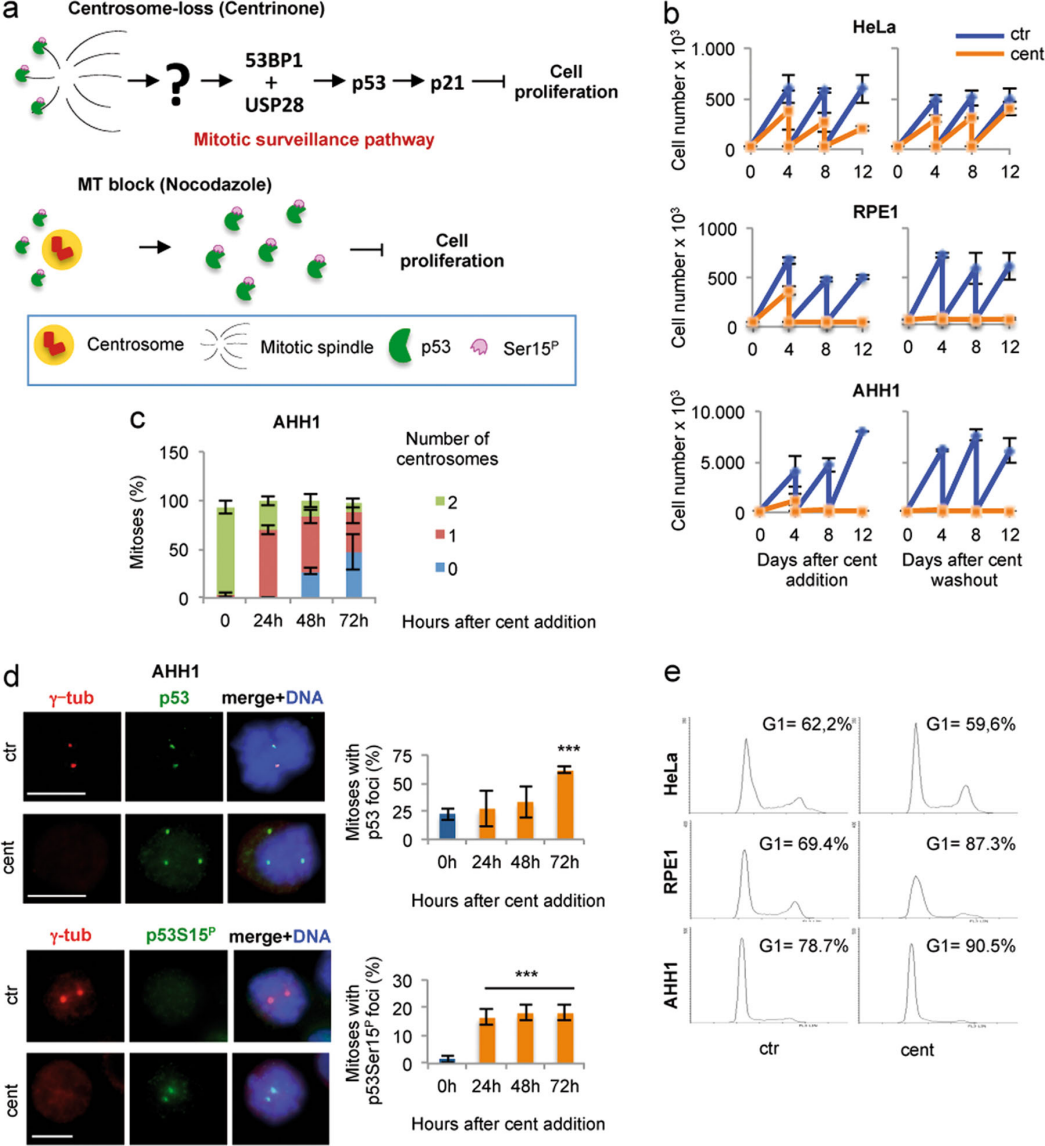
p53-MCL as a centrosome-associated sensor. **a** Upper panel: schematic representation of the mitotic surveillance pathway^16^ activated by centrosome-loss induced by centrinone treatment^15^. Lower panel: schematic representation of the accumulation of the p53Ser15^P^ foci induced by inhibition of MT dynamics by nocodazole-treatment^23^. **b** Cell passaging assays were performed with the indicated cells after the addition of DMSO (ctr) or centrinone (cent) and after their washout. HeLa and RPE1 cells behaved as reported^15^. The nontransformed AHH1 LCL irreversibly arrested after centrinone treatment as well as RPE1 cells. **c** Time course analysis of centrinone-induced centrosome-loss in AHH1 mitotic cells. The percentage of mitoses with 0, 1, and 2 centrosomes has been calculated by analyzing at least 50 mitoses per time point. **d** Representative IF images of ctr and cent-treated AHH1 cells (48 h time point) obtained by double IF for γ-tubulin/p53 and γ-tubulin/phosho-p53Ser15 show that centrosome-loss associated with the formation of extra-centrosomal p53 foci that can be phosphorylated at Ser15. Histograms show the percentages of mitotic cells with p53 and p53Ser15^P^ foci at the indicated time-points after centrinone addition. **e** The indicated cells, ctr or cent treated for 8 days, were analyzed for DNA content by flow cytometry. The relative cell-cycle profiles show that AHH1 cells accumulate in the G1 phase of the cell cycle. RPE1 and HeLa cells were used as controls and behaved similarly to previous report^15^

Centrosome fragmentation can be either a sign of defective centriole and centrosome components, or a generic consequence of mitotic fatigue, a condition resulting from mitotic delay of different origin^39,40^. Based on the usual checkpoint-related activities of p53, the mitotic delay we observed upon inhibition of p53-MCL, and the restricted presence of centrosome fragmentation in human nontransformed cells, we sought to test the hypothesis that, at each mitosis, p53 works as a centrosome-associated sensor. The recently described mitotic surveillance pathway, that arrests cell growth in response to centrosome-loss and prolonged mitosis in human nontransformed cells^14,15^, offers the opportunity to challenge our idea. We reasoned that, if human p53 must reach centrosomes, where it is dephosphorylated to allow cell-cycle progression^30^ (Fig. 1a), the absence of centrosomes should leave p53 devoid of its mitotic destination and activate the mitotic surveillance pathway (Fig. 6a, upper panel). This idea was further supported by our previous observations made upon inhibition of MT dynamics in LCLs from healthy donors (e.g., AHH1 LCL)^23^. In this condition, p53 did not reach the centrosomes, remained phosphorylated at Ser15 in discrete extra-centrosomal foci, and the cells arrested in the next G1 phase in a p21^WAF1^-dependent manner (Fig. 6a, lower panel)^23,30^. Because of the above considerations, we asked whether centrinone-induced centrosome-loss^15^ might also induce accumulation of discrete p53Ser15^P^ foci. Thus, AHH1 cells were treated with centrinone and analyzed for cell proliferation and centrosome-loss. The originally described RPE1 and HeLa cells were used as control^15^. In the presence of centrinone, AHH1 and RPE1 cells irreversibly arrested while HeLa tumor cells slowed-down, but reentered into the cell cycle upon centrinone washout, as previously reported (Fig. 6b). We then assessed the time of centrosome-loss and analyzed p53 subcellular localization by IF with both anti-p53 and anti-phospho p53Ser15 Abs. Starting from the 24 h time point, mitotic AHH1 cells showed reduction of centrosome number (Fig. 6c) that was associated with a significant accumulation of discrete, extra-centrosomal p53Ser15^P^ foci (Fig. 6d). Comparable results were obtained with RPE1 cells (Supplementary Fig. 3a) and HFs (data not shown) while, in HeLa tumor cells, p53 foci were not phosphorylated at Ser15 and their presence was independent of centrinone treatment (Supplementary Fig. 3b). Then, 4 days after centrinone treatment, the discrete p53 foci were no longer detectable and, similarly to the results reported by Wong et al.^15^, we observed accumulation in the G1 phase of the cell cycle (Fig. 6e). Thus, centrinone-induced centrosome-loss is associated with the formation of discrete p53Ser15^P^ foci.

### p53Ser15^P^ foci recruit 53BP1 to trigger the mitotic surveillance pathway

**Fig. 7:**
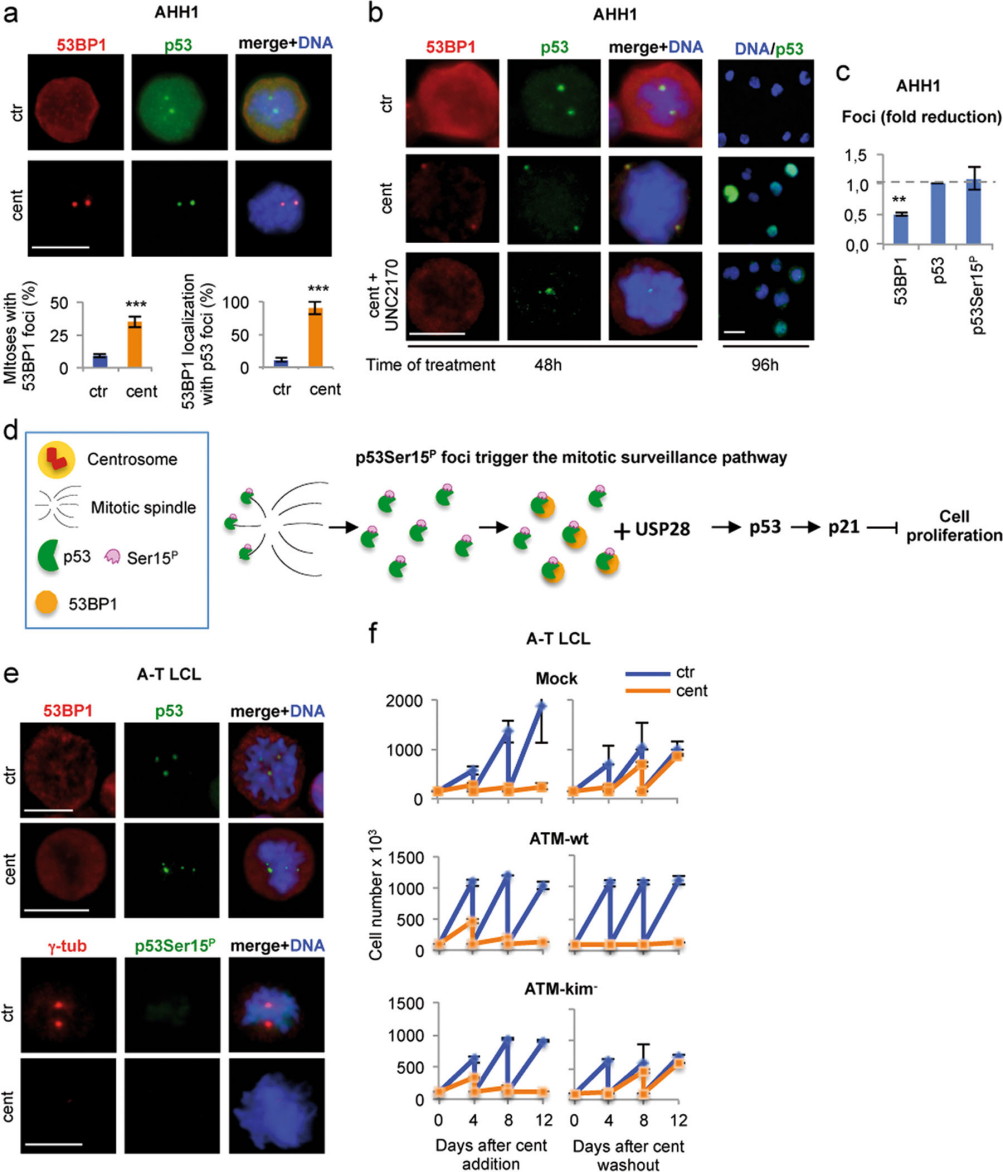
p53Ser15^P^ extra-centrosomal foci recruit 53BP1 and trigger the mitotic surveillance pathway. **a** AHH1 cells were treated with centrinone for 48 h and double immunostained with anti-p53 and anti-53BP1 Abs. Representative IF images show that in response to centrosome-loss, mitotic cells have extra-centrosomal p53 foci that colocalize with 53BP1 foci. Histograms report the percentages of mitotic cells carrying 53BP1 foci and in which the 53BP1 foci colocalize with the p53 foci. **b** AHH1 cells were treated, for the indicated times, with centrinone with or without the 53BP1 inhibitor UNC2170 and analyzed by IF for the indicated proteins. Representative IF images of the 96 h time point show that UNC2170 prevented the p53 nuclear staining induced by centrinone treatment, supporting an inhibitory activity of the 53BP1 function in the mitotic surveillance pathway. Representative IF images of the 48 h time point show that UNC2170 reduces the formation of 53BP1 foci but not extra-centrosomal p53 foci. **c** Quantification of the IF analyses performed in (**b**) or not shown in the images (i.e., p53Ser15^P^ foci) is reported in the histogram. The fold reduction induced by the addiction of UNC2170 on the indicated type of foci is shown. At least 30 mitotic cells per condition were analyzed. The results show that 53BP1 foci were significantly reduced while p53 and p53Ser15^P^ foci were unmodified suggesting that their presence is independent of 53BP1. **d** Schematic representation of p53 working as sensor for the mitotic surveillance pathway in human nontransformed cells. Upon centrosome-loss, the p53Ser15^P^ foci cannot reach the centrosome to be dephosphorylated (sensor shut down) to allow cell-cycle progression. Persistence of p53Ser15^P^ foci recruit 53BP1 and trigger the mitotic surveillance pathway. In this context, p53 acts both upstream (p53Ser15^P^ foci) and downstream (p53 transcription factor activating p21) the 53BP1/USP28 signaling axis. **e** To verify whether the ATM-dependent p53S15^P^ has a role in the formation of the 53BP1 foci, centrinone treatment and IF analyses were performed on ATM-defective cells (A-T LCL) as above. Representative IF images show that, after centrosome-loss, extra-centrosomal p53 foci were detectable but they were not phosphorylated at Ser15 and 53BP1 did not form foci that colocalize with the p53 foci. Scale bars, 10 µm. **f** Cell passaging assays were performed, as in Fig. 6a, with A-T LCL and with A-T LCL reconstituted with ATM-wt and a kinase defective mutant, ATM-kim^-^. Centrinone treatment of A-T LCL did not irreversibly arrest the cell cycle, as shown by cell proliferation after centrinone washout. However, expression of ATM-wt, but not ATM-kim^-^, restored the capacity of cells to irreversibly exit from the cell cycle

Previous data demonstrated that 53BP1 is required to induce irreversible cell-cycle arrest in response to centrosome-loss (Fig. 6a)^17–19^. Thus, we evaluated whether the p53 foci colocalize with 53BP1. After centrinone treatment, a significant number of discrete foci of 53BP1 appeared in both AHH1 (Fig. 7a) and RPE1 (Supplementary Fig. 4a) cells and double IF showed an overall colocalization with the p53 foci, suggesting a functional link between the two types of foci.

Next, we evaluated whether 53BP1 contributes to the formation of the p53 foci by treating AHH1 cells with both centrinone and the 53BP1 inhibitor UNC2170, that binds the 53BP1 tudor domains also involved in p53/53BP1 interaction^41^. Inhibition of 53BP1 reduced the formation of 53BP1 foci and the subsequent nuclear activation of p53 previously described in response to centrinone (Fig. 7b)^15^. However, we did not find any difference in the number of p53 foci or in their phosphorylation status at Ser15 (Fig. 7c) indicating that the presence of p53Ser15^P^ foci precedes 53BP1 recruitment and activation. Comparable results, i.e., accumulation of p53 foci in response to centrinone, were obtained with *53BP1*ΔRPE1 cells in which 53BP1 was knocked out by CRISPR/Cas9 (Supplementary Fig. 4b). These results indicate that centrosome-loss leaving p53 orphan of its mitotic centrosomal localization, promotes the formation of discrete foci of p53Ser15^P^ that, in turn, allow the recruitment of 53BP1 foci that triggers the mitotic surveillance pathway (Fig. 7d).

Finally, we asked whether ATM, which phosphorylates p53 at Ser15 and is necessary for p53-MCL, could be indeed involved in the phosphorylation of the p53Ser15^P^ foci required for 53BP1 recruitment/activation in response to centrosome-loss. ATM-defective cells from an A-T patient (A-T LCL) were treated with centrinone and analyzed by IF. We found that p53 foci were not phosphorylated at Ser15 and that 53BP1 did not colocalize with the p53 foci and remained dispersed (Fig. 7e). Consistent with this result, centrosome-loss did not irreversibly arrest A-T LCL that continued to proliferate after centrinone withdrawal (Fig. 7f, upper panel). Finally, the ATM-dependency was further confirmed by the fact that A-T LCL reconstitution with ATM-wt, but not with a kinase defective mutant, ATM-kim^−^, restored irreversibility of cell-cycle arrest (Fig. 7f, middle and lower panels). These results indicate that ATM-induced p53Ser15^P^ foci are required to recruit 53BP1 and to irreversibly arrest the cell cycle in response to centrosome-loss. Altogether, these data strongly support the hypothesis that the ATM-dependent p53-MCL works as sensor for the mitotic surveillance pathway.

### p53-MCL-defective cells accumulate numeral chromosome errors

The mitotic surveillance pathway is thought to prevent growth of cells that have an increased chance of making mitotic errors. Thus, we asked whether cells from patients carrying genetic alterations that impair p53-MCL might accumulate numeral chromosomal defects. Together with LCLs from A-T homozygous and heterozygous carriers, which have constitutive impairment of p53-MCL^29^, we analyzed LCLs from patients harboring heterozygous or homozygous mutations in the *PCNT* gene^42^. This gene encodes for pericentrin, a scaffold protein that anchors several centrosome factors and whose depletion provokes centrosome fragmentation^43^. By using *PCNT* depletion (PCNTi) as a positive control for centrosome fragmentation, we observed that PCNTi cells—both HFs and U2OS—exhibited a strong reduction of p53-MCL without activation of p53 stress response (Supplementary Fig. 5), suggesting that p53 belongs to the wide range of proteins directly or indirectly anchored to the centrosome through PCNT. In agreement, LCLs with homozygous and heterozygous *PCNT* mutations showed impairment of p53-MCL comparable to A-T LCLs (Table 1). Next, we analyzed the metaphase spreads of these LCLs for structural and numeral chromosome alterations. As shown in Table 1, no significant alterations of chromosome structure (gaps and breaks) were observed in any type of LCLs. In contrast, an increase of aneuploid metaphases were observed in those cells in which p53-MCL was defective, including RPE1 cells expressing HSPA9p, further supporting a link between p53-MCL and the mitotic surveillance pathway.

**Table 1:** p53-MCL-defective cells accumulate numeral chromosome abnormalities

Cell type	Mutation status	p53-MCL	CA^a^	AM^b^	<46	46	>46^c^
WT1 LCL	wild-type	91%	10	4.3	9	287	4
WT2 LCL	wild-type	84%	6	3.3	7	290	3
CV1559 LCL	PCNT htz	50%	8 ^ns^	9.3****	15	272	13
CV1576 LCL	PCNT hmz	3%	6 ^ns^	15.3****	32	254	14
K227RM LCL	PCNT hmz	8%	8 ^ns^	17****	35	249	16
665RM LCL	ATM htz	50%	3 ^ns^	12****	21	264	15
153RM LCL	ATM hmz	1%	3 ^ns^	16****	27	252	21
RPE1 ctr	wild-type	85%	1	3	6	291	3
RPE1 + HSPA9p	wild-type + HSPA9p	27%	4 ^ns^	22****	39	234	27

^a^Chromosome aberrations, i.e., gaps and breakes (*n* = 100 metaphases)

^b^Aneuploid metaphases (*n* = 100 metaphases)

^c^Metaphases with the indicated number of chromosomes (*n* = 300 metaphases)

^ns^not significant; ****p < 0.0001

## Discussion

Here, we have compared the centrosome-associated behavior of p53 in nontransformed human vs. human tumor or mouse cells and show that acute p53 depletion/deletion or selective impairment of p53-MCL induce centrosome fragmentation and cell death only in nontransformed human cells. Since p53 depletion/inactivation is commonly well-tolerated by tumor cells of different origin, as well as by normal mouse cells, the centrosome fragmentation was quite unexpected. Thus, we performed a large series of experiments that ruled out possible off-target or cell-specific effects and rescued centrosome fragmentation. In addition, we found that, at variance with its human ortholog which transiently moves to centrosomes in mitosis, mouse p53 is constitutively present at the centrosomes throughout the cell cycle in a MT- and ATM-independent manner. These observations highlight the existence of species-specific differences in p53 centrosome localization and function. Interestingly, these features resemble those of the recently described “centrosome-loss sensor” a human-specific, p53-dependent mitotic surveillance pathway whose activation irreversibly arrests nontransformed human cells, but not cancer cells or mouse cells^15^. In agreement, here we have provided evidence that human p53-MCL prevents the activation of the mitotic surveillance pathway and the subsequent p53-mediated cell-cycle arrest. In particular, we found that centrinone-induced centrosome removal, by hampering p53 centrosomal docking, triggers the accumulation of p53Ser15^P^ foci. These foci are able to recruit 53BP1 that, in turn, stabilizes p53. Thus, p53 triggers its own stabilization and further activation through the formation of ATM-dependent p53Ser15^P^ foci, so to induce irreversible cell-cycle arrest (Fig. 7d).

These results might appear incongruous with a few observations made in response to centrosome-loss^17–19^ which would argue against an ATM contribution in the mitotic surveillance pathway. However, a few aspects might reconcile these apparent divergences. (1) The screen method: 53BP1/USP28, but not ATM, have been identified as the proteins required to activate p53 upon centrosome-loss by selecting cells that still proliferate in the absence of centrosomes^17–19^. Our experiments showed that A-T cells reenter into the cell cycle upon centrinone withdrawal but do not proliferate in the presence of centrinone. This suggests that ATM-deficient clones cannot be selected. (2) The absence of p53 post-translational modification. In agreement with the non-canonical p53 activation described in the mitotic surveillance pathway, the ATM-p53 axis involved in p53-MCL is independent of DNA-damage response, while requires the dephosphorylation of p53Ser15^P^ as soon as it reaches the centrosomes^23^. The permanence of these discrete p53Ser15^P^ foci within the cytoplasm can be visualized at the single cell level by IF, but not necessarily detected at the whole cell-population level by WB. This would explain the reported absence of p53 post-translational modifications by WB^15^. (3) Centrosomal p53: the possibility that p53 might activate the mitotic surveillance pathway through its centrosomal localization has been taken into consideration by Lambrus and coauthors^19^, but excluded because they did not detect centrosomal p53. However, they analyzed interphase RPE1 cells, while p53 can be detected at the centrosome only during mitosis.

The mitotic surveillance pathway is believed to prevent the growth of cells undergoing mitotic errors triggered by prolonged mitosis and centrosome-loss^16^. Our results strongly support a role for p53-MCL in sensing these defects and triggering the activation of this pathway. Consistent with this model, we observed an accumulation of aneuploidy in p53-MCL defective LCLs, such as those carrying genetic alterations in the *ATM* and *PCNT* genes. Altogether, our data suggest that the ATM-p53 axis, in addition to its DNA caretaker activity, contributes to ploidy preservation by a centrosome-associated function.

## Materials and methods

### Cell culture and drugs

Human hTERT-immortalized dermal fibroblasts (HFs) (kindly provided by F. Loreni), MEFs, RPE1, HeLa, U2OS, HCT116, MCF.7, RKO, ZR75.1, ASC, HUVEC, SAOS-2, H1299 cells were cultured in DMEM GlutaMAX (Invitrogen); U87MG, A549, LoVo, PBMCs, LCLs and 32D cells were cultured in RPMI-1640 GlutaMAX (Invitrogen). The following LCLs were employed: AHH1, WT1, and WT2 from healthy donors; A-T 665RM mutant *ATM* heterozygous, A-T 153RM mutant *ATM* homozygous; A-T L6 ATM-null reconstituted with flagATM-wt or flagATM-kim^−^ mutant^44^ (kindly provided by D. Barilà); CV1559 mutant *PCNT* heterozygous; CV1576 and K227RM mutant *PCNT* homozygous. Media were supplemented with 10% heat-inactivated fetal bovine serum, 100 U ml^−1^ penicillin and 100 g ml^−1^ streptomycin (all from Invitrogen). CSC cells were cultured in serum-free medium. All the cell lines used were mycoplasma free. During live cell imaging, cells were cultured in DMEM medium without phenol red (Invitrogen). Clonogenic growth was calculated 12 days post-transfection and selection with G418 1 mg/ml (Thermo) by measuring the density of cells stained with crystal violet (5% in methanol, Sigma) for 10 min and analyzed by IMAGEJ. For cell synchronization, adherent cells at 30% confluences were grown in the presence of 2 mM thymidine for 18 h. After the first block, thymidine was removed and cells grown in fresh medium for 9 h for cell-cycle release. The second block follows the release by the addition of 2 mM thymidine and cultivation for 17 h. Synchronization in mitosis was assessed by DNA staining with HOECHST-33342 (Sigma) and IF analysis.

The following drugs were used at the indicated concentrations: 0.6 µM for 20 h or 10 µM for 10 min of nocodazole (Santa Cruz Biotechnologies); 2 µM cytochalasin B (Santa Cruz Biotechnologies); 10 µM KU-55933 (Sigma); 70 µM UNC2170 (Xcess Biosciences Inc.); 125 nM centrinone (MedChem); 5 µM CDDP (Sigma), 2 mM thymidine (T1895, Sigma); 0.6 µM Adriamycin (doxorubicin, Sigma); 1 µM 5-Azacytidine (Sigma). Control experiments were performed using the solvent DMSO (Sigma Aldrich).

### Plasmids and transfections

The following plasmids were used: pCAG-p53 carrying mouse wt-p53^45^; pLp53SP carrying human wt-p53^46^; pcDNA3-p53His175 carrying human p53H175R mutant^47^; pcDNA3.1-HSPA9^253–282^-His produced by cloning the p53 binding region of HSPA9 (aa 253–282) with restriction enzymes BamH1/Age1, in frame with the His6-tag in the pcDNA3.1V5-His-TOPO/lacZ and sequenced; pCAG3.1-p53-Δ(325–355) produced by cloning the human p53-Δ(325–355) (Addgene) into the mammalian pCAG3.1 vector using BamH1/EcoR1 restriction enzymes. Cells were transfected using Lipofectamine LTX and PLUS reagent (Life Technologies) according to the manufacturer’s instructions.

### Western blotting

Total cell extracts and purified centrosomes were prepared in lysis buffer [50 mM Tris-HCl (pH 8), 600 mM NaCl, 0.5% sodium deoxycholate, 0.1% SDS, 1% NP40 and 1 mM EDTA] supplemented with protease-inhibitor mix (Roche) and Halt Phosphatase Inhibitor Cocktail (Thermo Scientific). Proteins were resolved by SDS-PAGE using NuPAGE® Novex Bis-Tris Gels (Invitrogen), transferred onto nitrocellulose membranes (Bio-Rad), and analyzed with the required Abs. Immunoreactivity was detected by ECL-chemoluminescence (Amersham).

### Immunofluorescence

Cells were seeded onto poly-L-lysine coated coverslips, pre-permeabilized at RT in 0.25% Triton X-100 in PBS for 2 min, fixed in 2% formaldehyde for 10 min, washed in PBS, permeabilized in 0.25% Triton X-100 in PBS for 5 min, refixed and permeabilized in ice-cold Methanol at −20 °C for 10 min. For non-adherent cells, the pre-permeabilization step was avoided to preserve higher number of mitosis on the coverslips. Next, cells were blocked in 0.2% Triton X-100, 3% BSA in PBS for 30 min in a humidified chamber before applying the required Abs. DNA was marked with HOECHST-33342 (Sigma). Cells were examined by Olympus BX53 microscope with epifluorescence; photographs were taken (×100 objective) with a cooled camera device (ProgRes MF).

### Antibodies

For IF, the following Abs were employed: mouse monoclonal anti-p53 (DO.7, 1:300; DAKO Cytomation), (PAb421, 1:100; Calbiochem); rabbit polyclonal anti-phospho-p53Ser15 (1:50; Cell Signaling); rabbit monoclonal anti-phospho-p53Ser15 (D4S1H, 1:50; Cell Signaling); mouse and rabbit anti-γ-tubulin (1:500 and 1:1000, respectively; Sigma), rabbit polyclonal anti-centrin-2 (N-17-R, 1:800; Santa Cruz Biotechnology), mouse monoclonal anti-γ-tubulin-Cy3 (1:300; Sigma), rabbit polyclonal anti-53BP1 (1:100; Novus Biologicals); secondary 488- or 594-conjugated Abs (1:400, Alexa-Fluor). For WB, the following Abs were employed: rabbit polyclonal anti-PCNT (1:500; Abcam); mouse monoclonal anti-phospho-ATM1981 (1:1000; Rockland) and anti-ATM (1:1000; Santa Cruz Biotechnology); rabbit polyclonal anti-p53 FL393 (1:500; Santa Cruz Biotechnology); rabbit polyclonal anti-mouse p53 (1:5000)^45^; mouse monoclonal anti-phospho-p53Ser15 (1:1000; Cell Signaling); rabbit polyclonal anti-p21^WAF1^ (1:800; Santa Cruz Biotechnology); rabbit polyclonal anti-PCNT (1:1000, Abcam); rabbit polyclonal anti-CEP131 (anti-AZA1, 1:5000; Abcam); mouse monoclonal anti-GAPDH (1:10,000; Santa Cruz Biotechnology); mouse monoclonal anti-HSP70 (1:1000; Thermo Fisher Scientific); mouse monoclonal anti-γ-tubulin (1:1000) and anti-actin (1:10,000; Sigma); mouse monoclonal anti-MDM2 (2A10; 1:1000); mouse monoclonal anti-Chk1 (1:400, Santa Cruz Biotechnology); HRP-conjugated goat anti-mouse and anti-rabbit Abs (Biorad).

### RNA interference and RT-PCR

RNA interference was obtained by using commercially available specific stealth mix of three RNAi sequences (Invitrogen) for human or mouse p53, human PCNT, human CEP131, and by universal negative control stealth RNAi Negative Medium GC Duplex (Invitrogen). Cell were transfected using RNAiMAX reagent (Invitrogen), according to the manufacturer’s instructions. Cellular RNA was isolated 48 h after siRNA transfection by using the RNeasy mini kit (Qiagen), reverse transcribed using MuLV reverse transcriptase and employed for PCR reactions with the GoTaq® DNA polymerase (Promega). Exogenous human HSPA9^253–282^ was amplified using a forward primer annealing on the pcDNA3.1 vector and a reverse primer annealing on the human HSPA9 gene: FW 5′-ACTGCTTACTGGCTTATCG-3′; REV 5′-CAAGGCCTGGTCAAAGT-3′. For GAPDH the following primers were used: FW 5′-TCCCTGAGCTGAACGGGAG-3′; REV 5′-GGAGGAGTGGGTGTCGCTGT-3′.

### CRISPR/Cas9 based gene-editing

CrRNAs, TracrRNA and HiFi Cas9 Nuclease 3NLS were from Integrated DNA Technologies (www.idta.com/CRISPR/Cas9) in their proprietary Alt-R format and transfected according to the manufacturer’s instructions. The sgRNA sequence employed for p53 knockout is ACTTCCTGAAAACAACG.TTCTGG^48^. The vector for the generation of CRISPR–Cas9-mediated loss-of-function of 53BP1 was generated using the lentiCRISPR version 2 backbone (a gift from Feng Zhang; Addgene plasmid number 52961) (PMID: 25075903). Oligonucleotides yielding the small guide RNA corresponding to the sequence CTGCTCAATGACCTGACTGA (PMID: 27432897) were cloned according to the Feng Zhang protocol available at http://www.addgene.org. RPE1 cells were incubated with lentiviral supernatants generated using the plasmid described above and were selected for 3 days using 500 ng/mL puromycin (Sigma Aldrich, P9620). To obtain monoclonal lines, cells were seeded in 96-well plates to a density of 0.2 cells per well and incubated for 3 weeks. Clones were further expanded and characterized for protein depletion by WB.

### Centrosome isolation

Centrosomes were isolated as described^49^. We modified the first part of the protocol in which MTs and actin cytoskeleton need to be disrupted to allow centrosome purification. Since prolonged nocodazole-treatment at 37 °C inhibits p53-MCL in human cells^23^, we empirically determined the shortest time and lowest dose of nocodazole compatible with preservation of p53-MCL. In brief, after cell synchronization, mitotic cells (3 × 10^6^) and a same number of interphase cells were incubated in the presence of 2 μM cytochalasin B and 10 μM nocodazole for 10 min in ice to disrupt MT and actin cytoskeleton without perturbing p53-MCL. Then, cells were lysed and poured, centrosome purified and analyzed by WB^49^.

### Live cell imaging

Cells were seeded in 35 mm dishes (81153, ibiTreat, Ibidi) and transfected as described above. Cells were observed under an Eclipse Ti inverted microscope (Nikon) using a Plan Apo 40X objective (Nikon). During the whole observation, cells were kept in a microscope stage incubator (Basic WJ Okolab) at 37 °C and 5% CO_2_. DIC images were acquired every 6 min over a 48 h period by a DS-QiMc camera and the NIS-Elements AR 3.22 software (Nikon). Image and video processing were performed with NIS-elements AR 3.22.

### Flow cytometry

Cells were collected after the indicated treatment, fixed in ice-cold 50% methanol and stored at 4 °C. For DNA content analysis, cells were permeabilized in PBS 0.1% Triton X, stained with propidium iodide (Sigma) and analyzed by EPICS XL (Beckman Coulter, Brea, CA, USA).

### Statistics

Data are expressed as mean ± SD. *P* values were derived from unpaired two-tailed *t*-tests using GraphPad Prism software or Fisher’s exact test when appropriate. *P* values < 0.05 were considered significant.

## Supplementary information

Supplementary Figure S5

Supplementary Figure and Video Legends

Supplementary Figure S1

Supplementary Figure S2

Supplementary Figure S3

Supplementary Figure S4

Video 1

Video 2

Video 3

Video 4

Video 5

Video 6

Video 7

Detailed attribution of authors
