# Supplementary figures and images for: p53 mitotic centrosome localization preserves centrosome integrity and works as sensor for the mitotic surveillance pathway
Source: Cell Death Dis. 2019 Nov 7;10(11):850. doi: 10.1038/s41419-019-2076-1 (PMC6838180; doi:10.1038/s41419-019-2076-1)

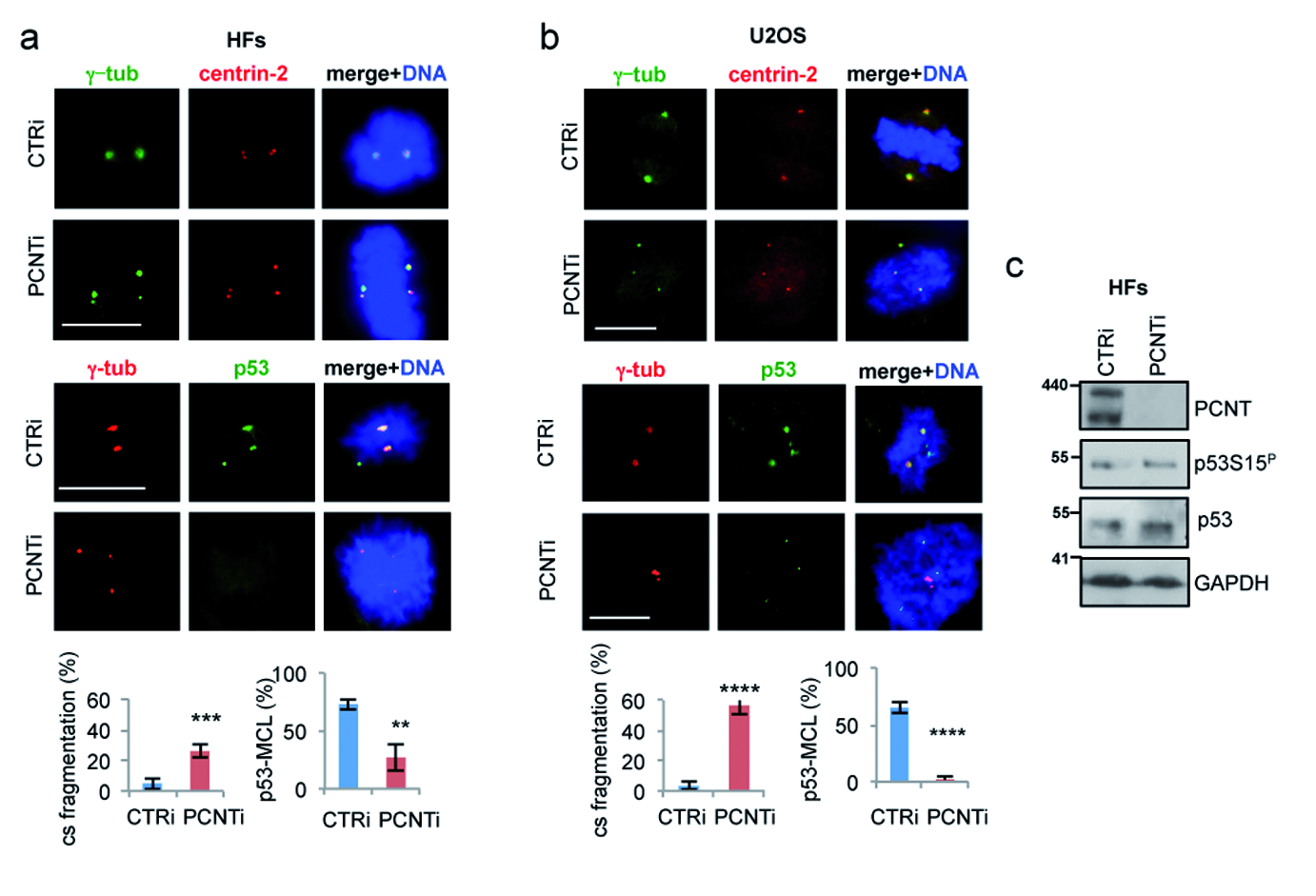

Supplement: Supplementary file 1 — Supplementary Figure S5 [file 41419_2019_2076_MOESM1_ESM.tif]

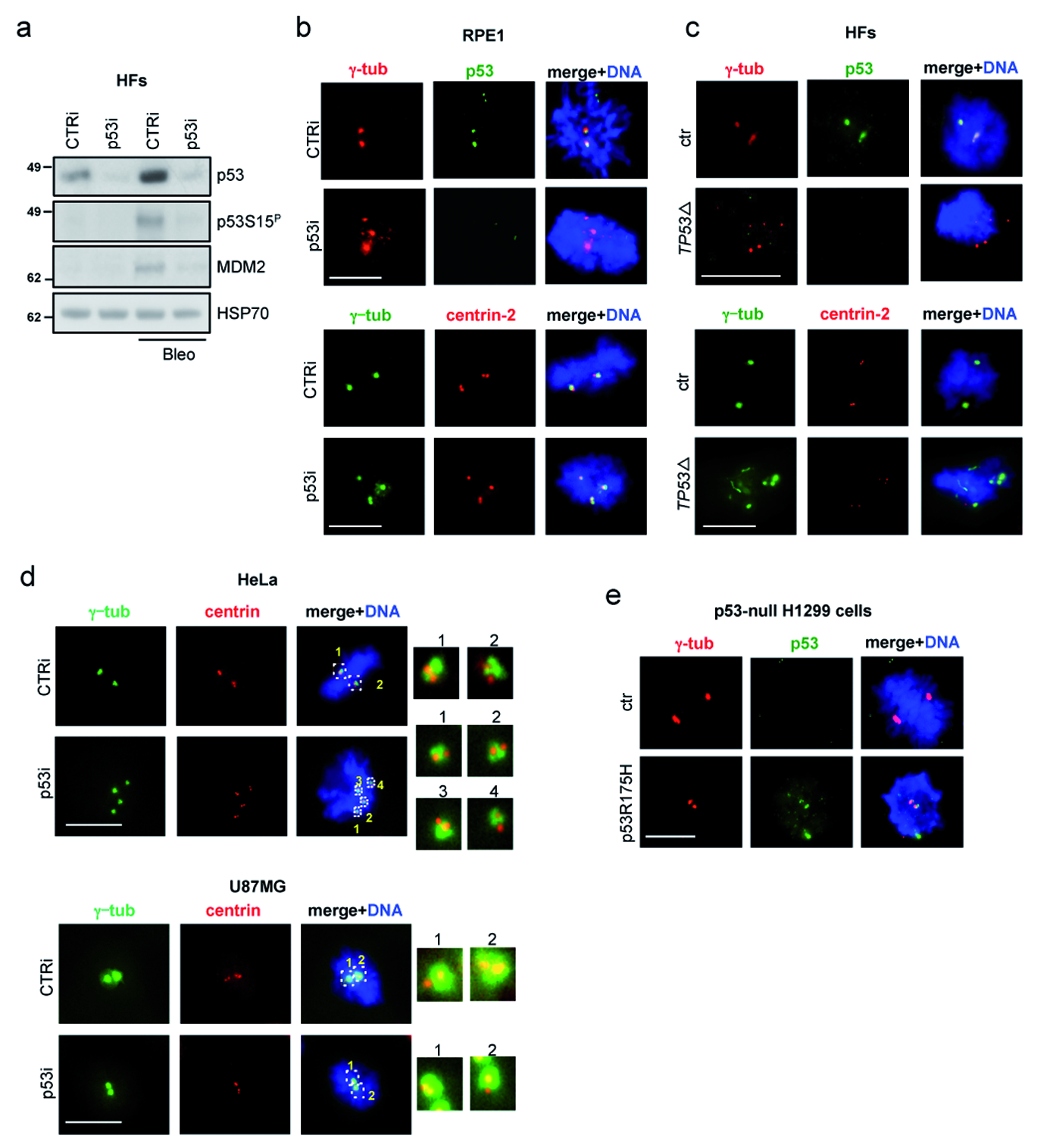

Supplement: Supplementary file 3 — Supplementary Figure S1 [file 41419_2019_2076_MOESM3_ESM.tif]

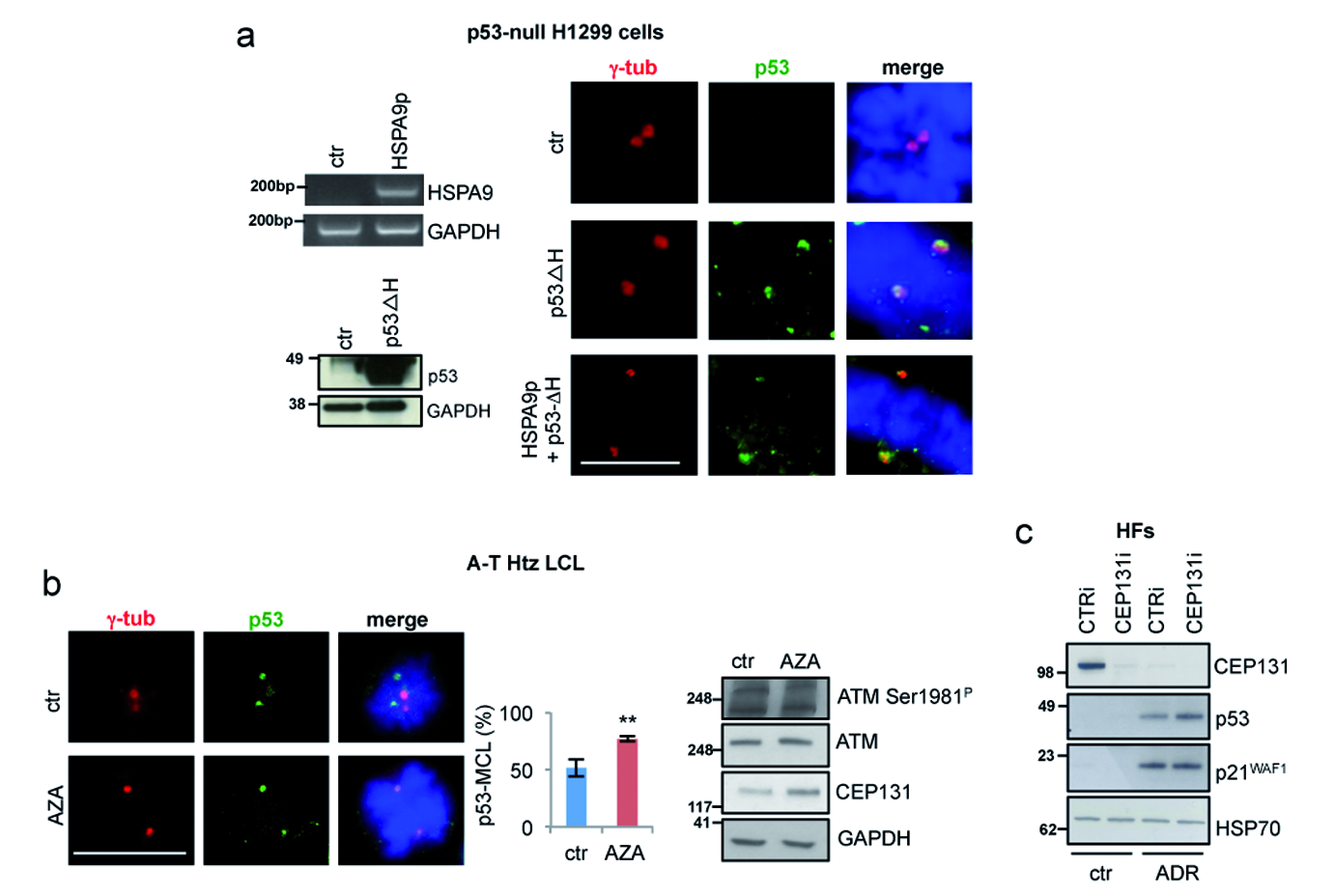

Supplement: Supplementary file 4 — Supplementary Figure S2 [file 41419_2019_2076_MOESM4_ESM.tif]

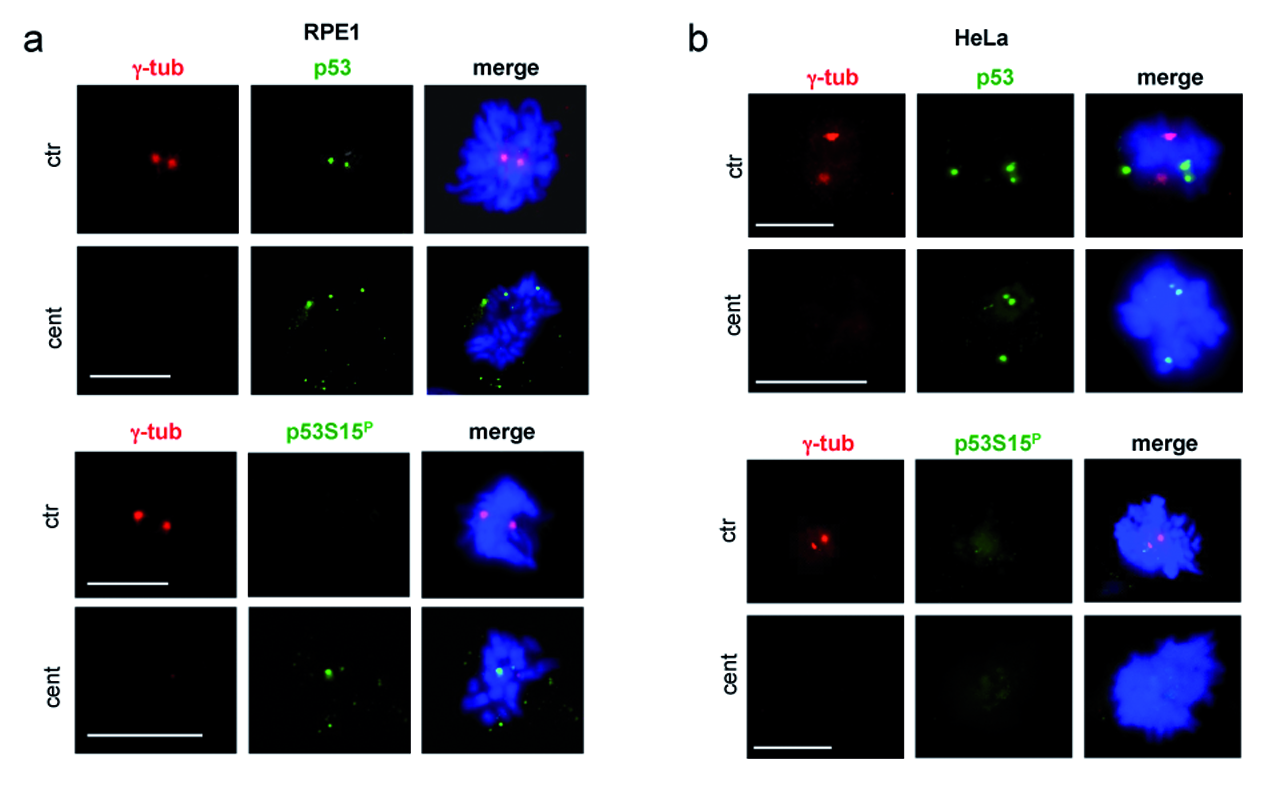

Supplement: Supplementary file 5 — Supplementary Figure S3 [file 41419_2019_2076_MOESM5_ESM.tif]

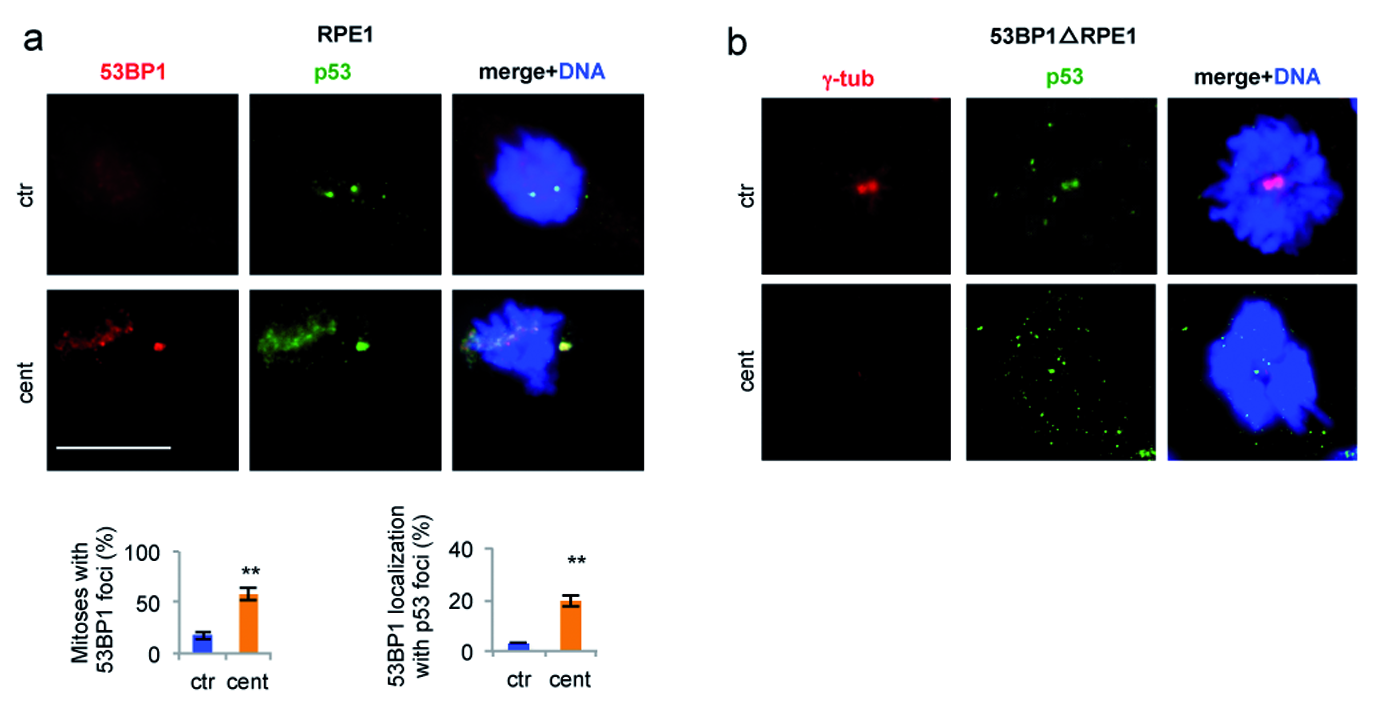

Supplement: Supplementary file 6 — Supplementary Figure S4 [file 41419_2019_2076_MOESM6_ESM.tif]
