# Supplementary material for: p53 mitotic centrosome localization preserves centrosome integrity and works as sensor for the mitotic surveillance pathway
Source: Cell Death Dis. 2019 Nov 7;10(11):850. doi: 10.1038/s41419-019-2076-1 (PMC6838180; doi:10.1038/s41419-019-2076-1)
Supplement: Supplementary file 2 — Supplementary Figure and Video Legends [file 41419_2019_2076_MOESM2_ESM.docx]

**Supplementary Figure and Video Legends**

**Figure S1.** Acute deletion of p53 induces centrosome fragmentation in human nontransformed cells, but not in tumor cells. **a** HFs transfected with CRTi and p53i siRNAs were analyzed for p53 activation by DDR with bleomycin treatment (Bleo). WBs for the indicated proteins and modifications show that p53i is sufficient to inhibit p53 stabilization/phosphorylation and MDM2 induction in response to bleomycin. **b** Representative double IF images of RPE cells in which endogenous p53 expression was depleted by p53i, whose quantification by WB is shown in Fig. 2d. **c** Representative double IF images of HFs in which endogenous p53 expression was depleted by transient CRISPR/Cas9 transfection (*TP53*Δ), whose quantification is shown in Fig. 2e. **d** The indicated cells were analyzed for centrosome number and structure by IF for γ-tubulin and centrin-2. Representative images with enlarged centrosomes or centrosomal material are reported. **e** Human p53-null cells (H1299) were transfected with an expression vector carrying the transcription-defective p53R175H mutant and the centrosomal localization of the mutant p53 was analyzed by IF. Representative images of p53R175H-MCL are shown and demonstrate the capacity of this p53 mutant to localize at the centrosome. Scale bars are 10 μm.

**Figure S2** p53-MCL impairment by HSPA9p and CEP131i. **a** To assess the ability of p53ΔH to localize at the centrosomes in the absence or presence of HSPA9p, p53-null H1299 cells were employed to avoid immunostaining of endogenous p53. H1299 cells were transfected with the p53ΔH expression vector with and without co-transfection of HSPA9p-expression vector**.** RT-PCR and WB analyses of the transfected products are shown**.** Representative IF images of p53 immunostaining show the localization of p53ΔH also in the presence of HSPA9p. **b** Representative images of A-T Htz LCL show that p53-MCL increases after treatment with 5-azacytidine (AZA). p53-MCL was analyzed by double IF for γ-tubulin/p53. The histogram shows the percentages of p53-MCL measured on mitotic cells (n=50 from each experiment). **P< 0.01. Scale bars are 10 μm. Immunoblots of the indicated proteins show that AZA increases CEP131 protein levels but not ATM protein or its phosphorylation at Ser1981. **c** CTRi and CEP131i HFs were treated with adriamycin (ADR) to activate the endogenous p53 protein and TCEs analyzed by WBs for the indicated proteins. No significant difference is present between CTRi and CEP131i in p53 and its target p21^WAF1^, indicating that CEP131i impairs p53-MCL but does not affect the stress-response function of p53.

**Figure S3** p53-MCL in the mitotic surveillance pathway. **a** RPE1 and **b** HeLa cells were treated with DMSO (ctr) or centrinone (cent), fixed and analyzed by IF for the indicated proteins. Representative IF images of the 48h time point show that, after centrosome loss, p53 formed extra-centrosomal foci in both cell types, but they are phosphorylated at Ser15 only in the RPE1 cells. In contrast, HeLa tumor cells show a constitutive presence of extracentrosomal p53 foci which are non phosphorylated at Ser14 both in the absence or presence of centrinone. Scale bars are 10 μm.

**Figure S4** p53-MCL in the mitotic surveillance pathway. **a** RPE1 cells were treated with centrinone for 48hr and double immunostained with anti-p53 and anti-53BP1 Abs. Representative IF images show that in response to centrosome-loss, mitotic cells have extra-centrosomal p53 foci that colocalize with 53BP1 foci. Histograms report the percentages of mitotic cells carrying 53BP1 foci and in which the 53BP1 foci colocalize with the p53 foci. **b**. 53BP1ΔRPE1 cells were treated with centrinone and assessed for the formation of p53 foci. Representative IF images of the 48h time point show the presence of p53 foci despite 53BP1 deletion supporting the data obtained with the 53BP1 inhibitor and reported in Fig. 7b.

**Figure S5** p53-MCL in PCNT-depleted cells **a** HFs and **b** U2OS cells were transfected with CTRi and PCNTi-specific siRNAs and immunostained with anti-γ-tubulin and anti-centrin-2 Abs to evaluated centrosome number and structure and with anti-γ-tubulin and anti-p53 Abs to evaluate p53-MCL. Representative IF images and related quantifications show a significant reduction of the p53 centrosomal localization induced by PCNT-depletion in both cell types. **c** CTRi and PCNTi HFs were analyzed by WB for the indicated protein to verify whether PCNT depletion per se induces p53 activation. No significant difference was observed between CTRi and PCNTi HFs in the total amount of p53 and in its Ser15^P^, two markers of p53 activation.

**Video 1** CTRi nontransformed HFs. Representative cell enters in mitosis and completes cell division in about 1hr 40min. For this and the following videos, phase-contrast images were captured every 6 min and the display rate is one frame every 250 ms. Indicated are bar (10 μm) and time. Still images of this video are shown in Figure 5a, upper panels.

**Video 2** p53i nontransformed HFs. Representative cell enters in mitosis and undergoes to mitotic catastrophe in about 2 hrs. Still images of this video are shown in Figure 5a, lower panels. Indicated are bar (10 μm) and time.

**Video 3** CEP131i nontransformed HFs. Representative cell enters in mitosis and undergoes to cell death in about 1hr 18min. Indicated are bar (10 μm) and time.

**Video 4** HSPA9p-expressing nontransformed HFs. Representative cell enters in mitosis and undergoes to cell death in about 7 hrs. Indicated are bar (10 μm) and time.

**Video 5** CTRi U2OS tumor cells. Representative cell enters in mitosis and completes cell division splitting in two daughter cells. Indicated are bar (10 μm) and time.

**Video 6** p53i U2OS tumor cells. Representative cell enters in mitosis, fails to divide and becomes a binucleated cell. Indicated are bar (10 μm) and time.

**Video 7** HSPA9p-expressing U2OS tumor cells. Representative cell enters in mitosis and completes cell division in about 1hr 25min. Indicated are bar (10 μm) and time.
