# Supplementary material for: p53 mitotic centrosome localization preserves centrosome integrity and works as sensor for the mitotic surveillance pathway
Source: Cell Death Dis. 2019 Nov 7;10(11):850. doi: 10.1038/s41419-019-2076-1 (PMC6838180; doi:10.1038/s41419-019-2076-1)
Supplement: Supplementary file 14 — Detailed attribution of authors [file 41419_2019_2076_MOESM14_ESM.pdf]

DECLARATION OF CONTRIBUTIONS TO ARTICLE

ADMC

Manuscript Number: CDDIS-19-2570

Journal Name: *Cell Death & Differentiation* (the 'Journal')

Proposed Title of the Contribution:

p53 mitotic centrosome localization preserves centrosome integrity and works as sensor for the mitotic surveillance pathway (the 'Contribution)

Author(s):

Claudia Contadini1, Laura Monteonofrio1*, Ilaria Virdia1*, Andrea Prodosmo1, Davide Valente1, Luciana Chessa2, Antonio Musio3, Luca L. Fava4, Cinzia (the 'Authors')

For all *CDD* articles, each person named as an author in the published version must be able to show he or she has contributed substantially to the article.

Authorship credit should be based on 1) substantial contributions to conception and design, acquisition of data, or analysis and interpretation of data; 2) drafting the article or revising it critically for important intellectual content; and 3) final approval of the version to be published. Authors should meet conditions 1, 2 and 3.

Any person who cannot be shown to have made a substantial contribution to the article cannot be listed as an author in the final version. The name of any person who is deemed to have made a minor contribution can, however, appear in the Acknowledgments section of the article.

Please complete the table below to indicate the contributions of all named authors to the manuscript.

Author Full Name:	Specification of Contribution to the Manuscript:
Claudia Contadini	planned and carried out the experiments, contributed to analyze the data and prepared the figures
Laura Monteonofrio	planned and carried out experiments on mouse cells and contributed to analyze the data
Ilaria Virdia	planned and carried out experiments on tumor and RPE1 cells
Andrea Prodosmo	planned and carried out the experiments on PCNT
Davide Valente	carried out time-lapse analyses
Luciana Chessa	performed genetic counseling, collected and analyzed LCLs
Antonio Musio	planned and carried out the experiments on PCNT and on karyotypes
Luca L. Fava	generated CRISPR – Cas9-mediated stable depleted cells and [illegible]
Cinzia Rinaldo	generated the HSPA9p vectors, supervised imaging experiments, contributed to analyze the data
Giuliana Di Rocco	supervised the work performed in tumor cells, revised the manuscript
Silvia Soddu	planned and coordinated the project, analyzed the data and wrote the manuscript

Please complete the table below to indicate the contributions of all named authors to the figures.

Figure 1:

Claudia Contadini
Ilria Virdia
Table 1
Andrea Prodosmo
Antonio Musio

Figure 2:

Claudia Contadini
Ilaria Virdia

Figure 3:

Claudia Cotadini
Cinzia Rinaldo
Ilaria Virdia

Figure 4:

Laura Monteonofrio
Andrea Prodosmo
Claudia Contadini

Figure 5:

Davide Valente
Claudia Contadini
Cinzia Rinaldo

Figure 6:

and Figure 7
Claudia Contadini
Ilaria Virdia
Luca Fava

Signed for and on behalf of the Author(s):	Print Name:	Date:
Silvia Soddu	Silvia Soddu	October 4th 2019
